# Role of Anti-GD_2_ Targeted PEG‑*b*‑PLGA Nanoparticles in the Treatment of MYCN Driven Neuroblastoma

**DOI:** 10.1021/acsabm.5c01709

**Published:** 2026-04-02

**Authors:** Ozde Gokbayrak, Derya Ozel, Ayca Tuncel, Fatma Yurt, Hatice Efsun Kolatan, Aylin Erol, Efe Ozgur Serinan, Tekincan Aktas, Osman Yilmaz, Safiye Aktas

**Affiliations:** † Department of Basic Oncology, Institute of Oncology, 37508 Dokuz Eylul University, Izmir 35340, Turkey; ‡ Department of Nuclear Applications, Institute of Nuclear Sciences, 37509 Ege University, Izmir 35100, Turkey; § Department of Laboratory Animal Science, Institute of Health Sciences, Dokuz Eylul University, İzmir 35340, Turkey

**Keywords:** neuroblastoma, MYCN, targeted drug delivery, AURORA inhibitors, anti-GD_2_

## Abstract

Neuroblastoma (NB) is an embryonic tumor originating from the neural crest. The most well-defined genetic alteration in NB is the overexpression of the MYCN protein. PI3K/AKT/mTOR and AURORA signaling pathways play a role in MYCN stabilization, and abnormal activation of these pathways has been identified in NB. Nanoparticle (NP) drug delivery systems targeting tumor cells directly assist in the combined delivery of agents and reducing toxicity. In this study, the aim was to develop NPs that reduce the activity of mTOR and AURORA pathways, potentially decreasing MYCN protein enhancement and stability, by combining inhibitors and targeting them specifically to the tumor, and to demonstrate their effect in NB. Everolimus (EVER) and tozasertib (TOZA) encapsulated in NP and targeted with dinutuximab β (DTX-β). Experiments including viability, apoptosis, gene and protein expression determination were performed. DTX-β/EVER + TOZA@PEG-*b*(*block*)-PLGA NPs were successful to reduce the cell viability and to increase apoptosis. In vivo studies demonstrated notable tumor growth inhibition without organ toxicity. Elevated caspase expression and suppressed proteins indicated enhanced apoptosis and reduced oncogenic activity. DTX-β/EVER-TOZA@PEG-*b*-PLGA may exert cytotoxic and apoptotic effects in NB. The use of targeted nanocarriers in NB treatment may enhance cytotoxic and apoptotic responses specifically in the tumor region.

## Graphical abstract

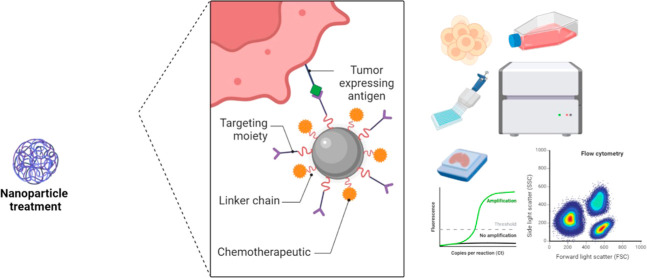

## 1 Introduction

NB is a tumor type of sympathetic nerve origin, characterized by high heterogeneity, with the ability to exhibit localized, spontaneously regressing, or widely metastasizing properties.^1^ Despite intensive protocols and alternative treatment approaches in advanced-stage disease, the two-year survival rate only reaches around 30–40%.^2^ The most well-defined genetic alteration in NB, occurring in approximately 20% of all NB cases and associated with a high-risk phenotype, is the overexpression of the MYCN protein.^3,4^ The MYCN protein controls cellular activities such as cell growth, division, and metabolism and is highly active in rapidly dividing malignant cells, accounting for approximately 70%. Therefore, MYCN protein is a targeted molecule for antitumoral activity.^5,6^ Direct targeting of MYCN protein is challenging due to the lack of suitable surfaces where drugs can bind on the DNA-binding domain.^7^ However, indirect targeting has been shown to be the most effective approach to inhibit or regulate MYCN protein.^8^ It is known that NB is dependent on several signaling pathways during its progression. Its association with the PI3K/Akt/mTOR signaling pathway results in an increase in MYCN protein. Studies on NB cells have shown that excessive PI3K signaling modulates GSK3β and mTOR, the regulated targets of AKT protein, and extends the half-life of MYCN protein.^9^ Therefore, PI3K/Akt/mTOR inhibitors are considered to be important potential drug targets for destabilizing MYCN protein (Chart 1).

**Chart 1:**
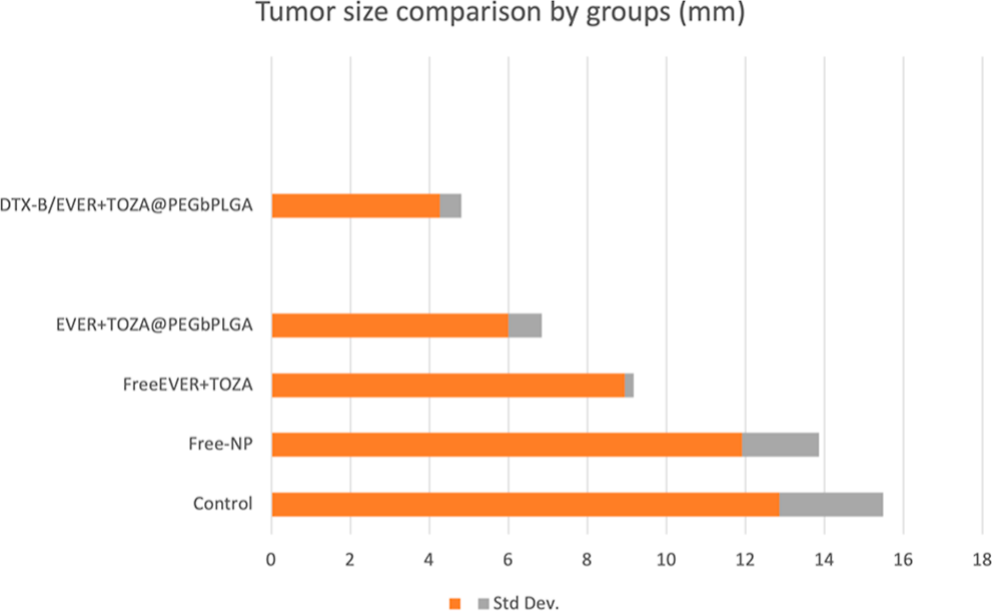
Tumor Size Comparison across Experimental Groups (mm)[Fn c1fn1]

a The dimensions of the tumors were provided in millimeters. Each group consisted of 7 mice (*n* = 7), and data are presented as mean ± SD. Statistical analysis was performed using the Kruskal–Wallis test followed by the Mann–Whitney *U* post-hoc test. All treatment groups showed significantly reduced tumor size compared with control (*p* < 0.05).

The protein kinase of the Aurora family, AURORA proteins, play an active role in the G2 and M phases of the cell cycle. The presence of AURORA increases the stability of MYCN and protects it from proteasomal degradation.^10^ Consequently, with high AURORA activity in MYCN-amplified diseases, it contributes to tumor cell proliferation.^11^ Therefore, suppression of AURORA activity is important for disrupting the stabilization of MYCN protein. The use of AURORA inhibitors in combination with chemotherapy is in the phase II stage of clinical trials.^12^

In cancer treatment, radiotherapy, chemotherapy and surgery are the main methods.^13^ However, conventional chemotherapy agents, in the classical sense, are not solely targeted to tumor cells within the system. Since they are distributed throughout the system, healthy cells are also exposed to the same cytotoxicity. This means that conventional chemotherapy agents exhibit side effects.^14^ Nanotechnology has made significant advances in both diagnosis and treatment. Over the past 25 years, research and clinical trials have shown promising results with nanosized chemotherapy agents and structures targeted specifically to tumors.^15^ Different NPs can carry different chemotherapy agents to the tumor site. Preclinical data from in vitro and in vivo studies with anticancer agents loaded into polymer NPs are highly promising.^16^ Polymer NPs exhibit strong structural stability and can be used to encapsulate high-capacity drugs.^17^ At the same time, modifying the surfaces of NPs allows specific transport of the drug to the tumor site and cellular internalization.

Polyethylene glycol–polylactic-*co*-glycolic acid (PEG–PLGA) NPs have emerged as a highly efficient drug delivery system due to their unique structural and functional properties. The combination of PEG and PLGA imparts several critical advantages, making them ideal for various therapeutic applications. One of the key benefits is their ability to enhance drug solubility and stability, which is crucial for hydrophobic drugs that are otherwise difficult to deliver effectively. Moreover, the biocompatibility and biodegradability of PLGA ensure that these NPs are safe for in vivo applications, gradually degrading into nontoxic byproducts easily eliminated from the body.^18^ The PEG component provides a “stealth” characteristic, significantly reducing opsonization and subsequent clearance by the mononuclear phagocyte system. This results in prolonged circulation times, allowing for more effective drug accumulation at the target site.^18–20^ Additionally, PEGylation improves the NPs’ ability to evade the immune system, further enhancing their potential for targeted drug delivery.^19,20^ This stealth property is particularly advantageous in cancer therapy, where it can facilitate the enhanced permeability and retention (EPR) effect, leading to better tumor targeting and reduced systemic toxicity. Furthermore, the versatility in synthesizing and functionalizing PEG–PLGA NPs allows for incorporating various targeting ligands, such as antibodies or aptamers. This capability enhances the specificity of drug delivery, ensuring that the therapeutic agents are delivered directly to the diseased cells while minimizing impact on healthy tissues.^21^

The expression of the disialoganglioside (GD_2_) antigen on the cell membrane of NB tumor cells is frequently observed.^22^ Regardless of the stage in NB, GD_2_ antigen is highly expressed in all primary tumors.^23,24^ DTX-β, approved by the US Food and Drug Administration (FDA) in 2015, was included in the standard treatment for high-risk NB patients.^25^ Since GD_2_ is considered a protein specific to NB, its use as a vector for delivering a GD_2_ agent or a chemotherapy agent within an NP to the tumor region is being tested, and studies are ongoing.^26^

Despite recent advances, there is no approved therapeutic strategy that simultaneously disrupts the two major regulatory mechanisms governing MYCN stability in neuroblastoma: (i) mTOR-dependent control of protein synthesis and (ii) AURORA-A-mediated protection of MYCN from proteasomal degradation. EVER, an mTOR inhibitor, reduces MYCN translation and shortens its half-life, whereas TOZA, a pan-AURORA kinase inhibitor, promotes MYCN destabilization through enhanced proteasomal turnover. Therefore, combining these agents presents a mechanistically complementary approach to MYCN suppression. However, their systemic administration is limited by off-target toxicity and suboptimal tumor accumulation. Targeting the GD_2_ antigen—uniformly and abundantly expressed on neuroblastoma cells—offers a rational strategy to enhance tumor-specific delivery. PEG-*b*-PLGA nanoparticles provide a clinically relevant, biocompatible platform that enables coencapsulation of EVER and TOZA while allowing surface functionalization with dinutuximab β (DTX-β) for GD_2_-directed targeting. To our knowledge, no previous study has integrated mTOR and AURORA inhibition within an antibody-targeted PEG–PLGA nanoplatform for MYCN-driven neuroblastoma. This mechanistically informed and tumor-specific design represents the key novelty of the present work.

This study aimed to evaluate the therapeutic efficacy of PEG-*b*-PLGA NPs loaded with the cancer drugs EVER and TOZA and targeted to NB using DTX-β. By incorporating these drugs into PEG-*b*-PLGA NPs, we aimed to enhance their delivery and effectiveness. This evaluation was conducted both in vitro and in vivo.

## 2 Materials and Methods

PEG-*b*-PLGA copolymer (PEG average *M*_n_ 5000, PLGA average *M*_n_ 15,000, lactide 50:50) and acetonitrile purchased from Sigma. EVER (cat no. S1120) and TOZA (cat no. S1048) purchased from Selleckchem. Dimethylformamide (DMF) (cat no. 103053) purchased from Merck. DTX-β antibody was kindly provided by Dokuz Eylul University Pediatric Oncology Department. *N*-Hydroxysuccinimide (NHS) (cat no. 902241) and ethyl-(3-dimethyl aminopropyl)carbodiimide (EDC) (cat no. 39391) was purchased from Sigma. Bicinchoninic acid (BCA) assay was purchased from Thermo Scientific (cat no. 23225). MYCN antibody (cat no. ab16898) was purchased from Abcam. AURORA antibody (cat no. sc-373856) was purchased from Santa Cruz. Caspase 3 (cat no. ab184787), caspase 8 (cat no. ab25901) and caspase 9 (cat no. ab202068) antibodies were purchased from Abcam. For qPCR, Magic SYBR Mix (Procomcure) kit was used. For RNA isolation, HiPure RNA Mini Kit (Magen) kit was used. Also we used The OneScript Hot cDNA Synthesis Kit for cDNA isolation.

### 2.1 Drug Encapsulation

PEG-*b*-PLGA nanoparticles coloaded with EVER and TOZA were prepared using the solvent displacement method. Briefly, 25 mg of PEG-*b*-PLGA (PEG *M*_n_ 5000; PLGA *M*_n_ 15,000; 50:50) were dissolved in 2 mL acetonitrile to obtain a clear polymer solution. EVER and TOZA (10 mg each) were dissolved in 200 μL DMSO and added dropwise to the polymer phase under magnetic stirring. This step was performed carefully to ensure the homogeneity of the mix. Then, twice the total volume of water was added, and to stabilize the solution, DMF equal to half the added water volume was introduced. All mixing steps were performed under gentle magnetic stirring (400–500 rpm) to prevent shear-induced disruption, and all drug-handling steps were conducted in amber tubes to protect the compounds from light exposure. The organic phase was then introduced dropwise into 4 mL deionized water to induce spontaneous nanoparticle formation. The suspension was stirred for 4 h to allow complete solvent diffusion. Following nanoparticle formation, purification was performed by high-speed centrifugation at 14,000*g* (≈12,000 rpm) for 20 min at 4 °C using a fixed-angle rotor (Eppendorf FA-45-24-11, Germany). The supernatant was carefully removed, and the nanoparticle pellet was washed twice with deionized water under the same centrifugation conditions. No centrifugal filter unit was used during purification; nanoparticles were pelleted directly by centrifugation. All purification and wash steps were performed in triplicate (*n* = 3), and free drug quantification in the final wash confirmed complete removal of unbound EVER and TOZA. After the final wash, the pellets were resuspended in deionized water for subsequent characterization and drug-loading analyses. All purification steps were performed in triplicate (*n* = 3). The purified nanoparticles were lyophilized to obtain a dry, storage-stable powder.^19,27^

### 2.2 Preparation and Functionalization of EVER + TOZA@PEG-*b*-PLGA with DTX-β Antibody

Surface conjugation of DTX-β onto EVER + TOZA@PEG-*b*-PLGA nanoparticles was performed using EDC/NHS coupling chemistry. Briefly, 10 mg of nanoparticles were dispersed in 2 mL MES buffer (pH 5.5). Based on the calculated amount of terminal carboxyl groups on PEG-*b*-PLGA (1.25 μmol), EDC and NHS were added at molar ratios of COOH/EDC/NHS = 1:4:8, corresponding to 0.96 mg EDC (5 μmol) and 1.15 mg NHS (10 μmol). Activation and conjugation reactions were performed under gentle stirring without vortexing to avoid antibody denaturation, and all steps were protected from light. The mixture was stirred for 30 min at room temperature to allow formation of NHS-esters on the nanoparticle surface. Following activation, DTX-β (8 mg, 10 μmol) dissolved in PBS (pH 7.4) was added to the activated nanoparticles at a 1:1 molar ratio with NHS-esters. Conjugation was carried out for 2 h at room temperature under gentle stirring. The product was purified by centrifugation at 14,000*g* for 20 min at 4 °C, followed by two additional washes with deionized water to remove unbound DTX-β. Free antibody remaining in the final supernatant was assessed by BCA assay to confirm complete purification. The final DTX-β-functionalized nanoparticles were resuspended in nuclease-free water or lyophilized for storage.

### 2.3 Nanoparticle Characterization

For DLS measurement, the hydrodynamic diameter, polydispersity index (PDI), and zeta potential of EVER + TOZA@PEG-*b*-PLGA and DTX-β/EVER + TOZA@PEG-*b*-PLGA nanoparticles were determined using a dynamic light scattering (DLS) instrument equipped with electrophoretic light scattering capability (e.g., Zetasizer Nano ZS, Malvern Instruments, UK). Nanoparticles were dispersed in deionized water at a concentration of 0.5 mg/mL, vortexed for 30 s, and sonicated in a bath sonicator for 5 min (40 kHz, ∼100 W) to ensure a homogeneous dispersion. Measurements were performed at 25 ± 0.1 °C after an equilibration time of 120 s. The backscattering angle was set to 173°, assuming a refractive index of 1.59 for the polymer matrix and 1.33 for the dispersant. For each sample, three independent measurements were recorded, each consisting of 10–15 subruns, and the *Z*-average diameter and PDI were reported as mean ± standard deviation (SD, *n* = 3). Zeta potential was measured by electrophoretic light scattering using folded capillary cells, and the results were also expressed as mean ± SD (*n* = 3). All DLS measurements were carried out immediately after purification and before freeze-drying. Lyophilization was performed solely for storage and did not influence the size characterization reported in this study.

The morphology and surface characteristics of the nanoparticles were analyzed by scanning electron microscopy (SEM) using a field-emission instrument (e.g., JSM-7500F, JEOL, Japan). For specimen preparation, 10 μL of a dilute nanoparticle suspension (0.1 mg/mL in deionized water) was dropped onto aluminum stubs covered with carbon tape and allowed to air-dry at room temperature overnight. Dried samples were sputter-coated with a thin gold/palladium layer (∼5 nm) using a sputter coater (e.g., 25 mA, 60 s) to prevent charging. SEM images were acquired under high-vacuum conditions at an accelerating voltage of 5–10 kV and a working distance of 8–10 mm, using magnifications between 10,000× and 100,000×. Particle size was evaluated by measuring at least 100 particles per sample using ImageJ software, and the average diameter was reported as mean ± SD (*n* = 3 images per formulation).

FTIR spectra of EVER, TOZA, DTX-β and the nanoparticle formulations were recorded using an FTIR spectrometer equipped with an ATR (attenuated total reflectance) accessory (Spectrum Two, PerkinElmer, USA). Lyophilized samples were placed directly on the ATR crystal without any further preparation. Spectra were collected in the range of 4000–400 cm^–1^, at a spectral resolution of 4 cm^–1^, accumulating 32 scans per sample. Background spectra were acquired under identical conditions prior to each measurement. All spectra were subjected to automatic baseline correction and vector normalization using the manufacturer’s software to ensure comparability between samples. At least three independent FTIR measurements were obtained for each formulation.

### 2.4 Loading Capacity

The drug loading capacity and efficiency of EVER and TOZA loaded on the synthesized PEG-*b*-PLGA nanostructure were evaluated by UV–vis spectrophotometric analysis using a microplate reader. After the drug loading experiment, the EVER + TOZA@PEG-*b*-PLGA NP was centrifuged, and the free EVER and TOZA in the supernatant were stored to calculate the loading efficiency. Following nanoparticle formation, purification was performed by centrifugation at 14,000*g* for 20 min at 4 °C. The supernatant was carefully removed, and the nanoparticle pellet was washed twice with deionized water using the same centrifugation conditions (14,000*g*, 20 min). After the final wash, the pellets were resuspended in deionized water for subsequent characterization and drug loading analyses. The supernatant containing the free drugs was dissolved in PBS at pH 7.4 to maintain a consistent environment similar to that used in other stages of the study. This ensured that the solubility of the drugs was comparable to their saturated solubility in the dispersion medium. For the quantification of EVER and TOZA, UV spectra of the drugs were carried out at the characteristic absorbance peak at 279 nm for EVER and 253 nm for TOZA. A series of EVER and TOZA solutions were prepared at different concentrations and their absorbance values were determined at their characteristic wavelengths in a microplate reader. A concentration–absorption curve plot was prepared using the absorbance values obtained by UV spectrometry to measure drug loading. Once the amounts of agents had been determined by reference to the standard curve graph, the percentage drug loading efficiency was calculated according to the total amount of drug initially added and the amount of drug in the supernatant. According to the calibration curve, the loading capacity and encapsulation efficiency of NPs were obtained using the following formulas

Drugloadingcapacity(%):[(weightofinitiallyaddeddrugs−weightofunbounddrugsinthesupernatant)]/[weightoftheNPsobtained]×100

$$Drug\;loading\;efficiency\;\left(\%\;EE\right):\;\frac{amount\;of\;loaded\;EVER}{weight\;of\;initially\;added\;EVER}\times 100\;\frac{amount\;of\;loaded\;TOZA}{weight\;of\;initially\;added\;TOZA}\times 100$$

All loading capacity and encapsulation efficiency measurements were performed in triplicate (*n* = 3), and results are presented as mean ± standard deviation (SD).

### 2.5 Quantification of the Amount of DTX-β Bound to DTX-β/EVER-TOZA@PEG-*b*-PLGA NP

The amount of DTX-β bound to the DTX-β/EVER + TOZA@PEG-*b*-PLGA NP nanohybrid structures was determined using a BCA Kit (Pierce, Thermo Scientific). All washing and dilution buffers were prepared in accordance with the manufacturer’s instructions to ensure compatibility with the pH-sensitive BCA assay. For this purpose, a standard curve was generated with bovine serum albumin (BSA) to determine the DTX-β concentration in the supernatants by spectrophotometric analysis at 562 nm using a Thermo Scientific Multiskan microplate spectrophotometer (Japan). BSA was used for standard calibration in accordance with the manufacturer’s instructions. The concentration of free DTX-β in the supernatant of DTX-β/EVER + TOZA@PEG-*b*-PLGA NP was assessed by measuring the absorbance values of the samples and comparing them to the standard curve derived from BSA absorbance values (*y*) and concentrations (*x*). The concentration of free DTX-β in the supernatants was calculated by reference to the standard curve. The BSA calibration curve used for quantification is provided in the Supporting Information (Figure S1). The amount of DTX-β bound to the nanohybrids was then determined by subtracting the free DTX-β from the initially added DTX-β. DTX-β loading was expressed as μg of antibody per mg of dry EVER + TOZA@PEG-*b*-PLGA NP. All measurements were performed in duplicate. Binding efficiency (%) was calculated using the following equation

$$\%\;Binding\;efficiency=\left(\frac{initial\;DTX‐\beta -free\;DTX‐\beta }{initial\;DTX‐\beta }\right)\times 100$$

This method allowed a clear evaluation of the conjugation efficiency and provided an accurate measurement of the DTX- β bound to the nanohybrid structures.

### 2.6 In Vitro Drug Release Study

The in vitro release of EVER and TOZA from the DTX-β/EVER + TOZA@PEG-*b*-PLGA nanoparticles was evaluated at physiological pH (PBS, pH 7.4), consistent with the data presented in Section 3.3. Nanoparticles were dispersed at a final concentration of 1 mg/mL in 5 mL of PBS to ensure a sufficiently large medium volume and maintain sink conditions during the entire release period. Samples were incubated at 37 °C under gentle shaking (100 rpm). At predetermined time points (15 min, 30 min, 1 h, 2 h, 3 h, 4 h, 6 h, 24 h, 48 h, 72 h, and 96 h), 500 μL of the nanoparticle suspension was withdrawn and centrifuged at 14,000*g* for 20 min to separate the free drug in the supernatant from the nanoparticles. The supernatant was carefully collected for analysis, and an equal volume (500 μL) of fresh, prewarmed PBS (37 °C) was immediately added to the release medium to maintain constant volume and preserve sink conditions. EVER and TOZA concentrations were quantified using UV–vis spectrophotometry at their characteristic wavelengths (279 nm for EVER and 253 nm for TOZA), based on previously prepared calibration curves. Cumulative release (%) was calculated relative to the total initial drug content per milligram of nanoparticles. All experiments were performed in triplicate, and results are presented as mean ± standard deviation (SD).

### 2.7 In Vitro Assays

#### 2.7.1 Cytotoxicity Assay

KELLY cell line was cultured using RPMI 1640 medium supplemented with 1% l-glutamine 1% penicillin/streptomycin, and 10% FBS. The cultures were maintained in a humidified atmosphere containing 5% CO_2_ at 37 °C. After the cells reached 80% confluency, they were multiplied by passaging to 96-well plates, with 10^4^ cells in each well, and the MTT test was performed. Various doses of EVER and TOZA combination and NPs were applied. 10 μL of MTT solution was added to 100 μL of supernatant in each well of the plate. The cells were then incubated at 37 °C for 3.5 h. Subsequently, the plates were removed from the incubator for dimethyl sulfoxide (DMSO) washing. Finally, colorimetric readings were obtained at 590 nm using a microplate reader, and absorbance values were recorded. The control group was considered 100% viable, and the viability of the samples was normalized to the control.

#### 2.7.2 Flow Cytometry

The free drugs (EVER + TOZA, DTX-β), EVER + TOZA@PEG-*b*-PLGA, and DTX-β/EVER + TOZA@PEG-*b*-PLGA agents were applied to KELLY cells seeded in 6-well plates. The IC_50_ doses determined in the MTT assay were applied, and cell pellets were collected and treated with annexin V/PI staining. Apoptosis/necrosis ratios were determined using a flow cytometry device.

### 2.8 In Vivo Experiments

All animal experiments followed up to the ARRIVE guidelines and were performed in accordance with the National Research Council’s Guide for the Care and Use of Laboratory Animals (Eighth edition). All methods conducted received permission from the Dokuz Eylul University’s Local Ethics Committee for Animal Experiments (HADYEK) with approval protocol number 46/2019. Each experimental group consisted of 7 mice (*n* = 7). For in vivo results, statistical comparisons between groups were performed using the Kruskal–Wallis test followed by the Mann–Whitney *U* posthoc test. Following manual tumor size measurement, agents were administered to mice with tumor diameters up to 0.8 cm. Treatment was performed by daily intravenous administration for 5 days after the tumor was formed.^28^ Animals which were unable to continue the experiment for any reason (infection, drug reaction, general condition disorder, etc.) or died before the end of the experiments were excluded from the experimental and statistical scope. In order to generate a tumor, KELLY cells were injected into each group. The control group received only saline buffer. A free NP with no drug load was administered to the second group. Following the determination of the IC_50_ dose, the free forms of TOZA and EVER agents were mixed in the third group. The recommended dosage for free-EVER + TOZA is 250 μM. The fourth group received EVER + TOZA@PEG-*b*-PLGA treatment (250 μM of EVER + TOZA combination encapsulated into NP). The fifth group received DTX-β/EVER + TOZA@PEG-*b*-PLGA as the target treatment. The tumor’s size was measured, and the animals were sacrificed 2 days after the final dose of drugs were applied. Extracted from mice that were sacrificed included tumor tissue, lung, kidney, brain, heart, liver, and spleen. For analysis, samples were kept in 10% formalin. Following tissue fixation, paraffin was used to embed the blocks. Blocks were sectioned for histological analysis.

#### 2.8.1 Immunohistochemistry Protocol

IHC was performed after obtaining 5 μm-thick sections from the tumor tissue blocks of sacrificed mice.^29^ Briefly, the tissues were deparaffinized in pure xylene, washed in 96% alcohol for 3 × 5 min, and finally rinsed with distilled water. To inactivate the endogenous peroxidase enzyme, the sections were treated with 3% H_2_O_2_ in methanol for 10 min. After rinsing with distilled water, the antigen retrieval step was performed by incubating the sections in citrate buffer at pH 6.0 at 98 °C for 20 min, followed by cooling for 20 min. The sections were then washed in PBS for 3 × 5 min. Subsequently, primary antibodies (MYCN antibody; 1:200, AURORA antibody; 1:100, caspase 3-8-9 antibodies; 1:300, 1:400, 1:400) were applied to all sections and incubated overnight at room temperature in a humid chamber. The sections were then treated with a secondary antibody, followed by serial washes and stained with DAB for 5 min. After rinsing with water for 2 × 5 min, the sections were stained with hematoxylin for 2 min. Dehydration in a series of increasing concentrations of ethanol and clearing in xylene was performed, and the preparations were made ready for microscopy.

Protein expression levels were estimated by calculating the percentage of positively stained cells in immunohistochemically labeled tissue sections. Images were analyzed using **ImageJ** (**Fiji**) **software**. Color deconvolution was applied to separate DAB staining from hematoxylin counterstaining. A consistent threshold was applied across all images to distinguish positively stained cells, and the percentage of positive cells was calculated relative to the total number of nuclei (identified using hematoxylin stain). At least five random fields per group were analyzed.

#### 2.8.2 Histopathological Examination

To evaluate the safety of the nanoparticles in vivo, histopathological staining was performed on tumor tissue sections using the hematoxylin and eosin (H&E) method. For paraffin-embedded sections, deparaffinization was first performed by immersing the slides in xylene for 3 min, followed by two additional changes of xylene for complete removal of paraffin. The sections were then rehydrated through a graded series of ethanol concentrations, starting with two washes in 100% ethanol for 2 min each, followed by 95% ethanol for 2 min, and finally 70% ethanol for 2 min. After rehydration, the sections were rinsed in distilled water for 2 min. Next, the sections were stained with hematoxylin for 5–10 min to visualize nuclear structures. Excess stain was removed by rinsing the slides under running tap water for 5 min. If overstaining occurred, differentiation was achieved by briefly dipping the slides in acid alcohol, followed by another rinse in tap water for 2 min. To enhance nuclear staining (bluing), slides were optionally immersed in ammonia–water or Scotts tap water substitute for 30 s to 1 min and then rinsed again under running tap water for 5 min. Following hematoxylin staining, the sections were counterstained with eosin Y for 1–3 min to stain the cytoplasm. Excess eosin was removed by briefly rinsing the slides in distilled water. The stained sections were then dehydrated through a series of ethanol washes, beginning with 70% ethanol for 2 min, followed by 95% ethanol for 2 min, and finally 100% ethanol for two additional changes, each lasting 2 min. To complete the preparation, the sections were cleared in xylene for 30 min. Finally, the slides were mounted with a coverslip and allowed to dry. The stained tissue sections were then examined under a microscope to assess the morphology and cellular details, providing valuable insights into the histopathology of the tumor tissues.

#### 2.8.3 RNA Isolation from Tumor Tissue

Total RNA from tumor tissues was performed using the RNA isolation kit according to the manufacturer’s instructions. Tumor tissues were homogenized using a homogenizer device. Then, 1 mL of MagZol reagent was added to the tissues. RNA was separated with 200 μL of chloroform. Then, it was precipitated with an equal volume of isopropanol. After washing with ethanol, it was evaporated, and RNA was dissolved in RNase-free water. The quality and concentration of RNA were measured using a NanoDrop spectrophotometer.

#### 2.8.4 cDNA Synthesis

Briefly, using 10 μL of isolated RNA, cDNA synthesis was performed with primers and enzyme. The synthesis involved the following temperatures and time intervals: 25 °C for 10 min, 50 °C for 30 min, and 85 °C for 5 min.

#### 2.8.5 qPCR

qPCR was performed to determine the mRNA gene expression levels of Aurora A, B, C, and MYCN. Specific primers for the gene regions, forward and reverse primers, were used at a concentration of 10 μM. qPCR was performed by the 2× SYBR Mix kit. The relative expression levels of mRNAs were calculated using the 2^–ΔΔCt^ method and normalized by GAPDH.

### 2.9 Statistical Analyses

The data will be presented as mean ± standard deviation. Statistical analyses were performed using the SPSS 29.0 (IBM) software package at a significance level of *p* < 0.05. The conformity of the data to a normal distribution was assessed using the Kolmogorov–Smirnov test. Intergroup data were analyzed using the Kruskal–Wallis test, followed by the Mann–Whitney *U* test.

## 3 Results

### 3.1 Characterization of the Synthesized Nanoparticle

#### 3.1.1 The Amount of DTX-β Bound to DTX-β/EVER-TOZA@PEG-*b*-PLGA NP

After loading EVER and TOZA to PEG-*b*-PLGA NP, DTX-β was conjugated onto the EVER + TOZA@PEG-*b*-PLGA NPs, and the binding efficiency was determined using the BCA method. According to the BCA method, it was determined that 26.0 μg of DTX-β were present per 1 mg of dry EVER + TOZA@PEG-*b*-PLGA NP, corresponding to a binding efficiency of 52.16% based on an initial input of 500 μg DTX-β for 10 mg nanoparticles.

#### 3.1.2 Dimensional Analysis

The size and zeta potential measurements were performed using the DLS measurement method. The size measurement of the NPs was performed (Table 1). In addition, in Figure 1, the SEM and DLS images also present the morphological and dimensional analyses of EVER + TOZA@PEG-*b*-PLGA and DTX-β/EVER + TOZA@PEG-*b*-PLGA NPs. The SEM images show that the EVER + TOZA@PEG-*b*-PLGA NPs have a smooth and spherical structure. After lyophilization, no visible aggregation was observed, and redispersed nanoparticles retained their original size distribution and PDI, indicating that the freeze-drying process did not adversely affect colloidal stability.

**Table 1:** DLS Measurement Results of the NPs^a^

NP	*Z*-average (nm)	PDI	zeta potential (mV)
EVER + TOZA@PEG-*b*-PLGA	148.1 ± 7	0.369	–27.5 ± 2
DTX-β/EVER + TOZA@PEG-*b*-PLGA	169.7 ± 6	0.401	–41.1 ± 2

a PDI: polydispersity index.

**Figure 1:**
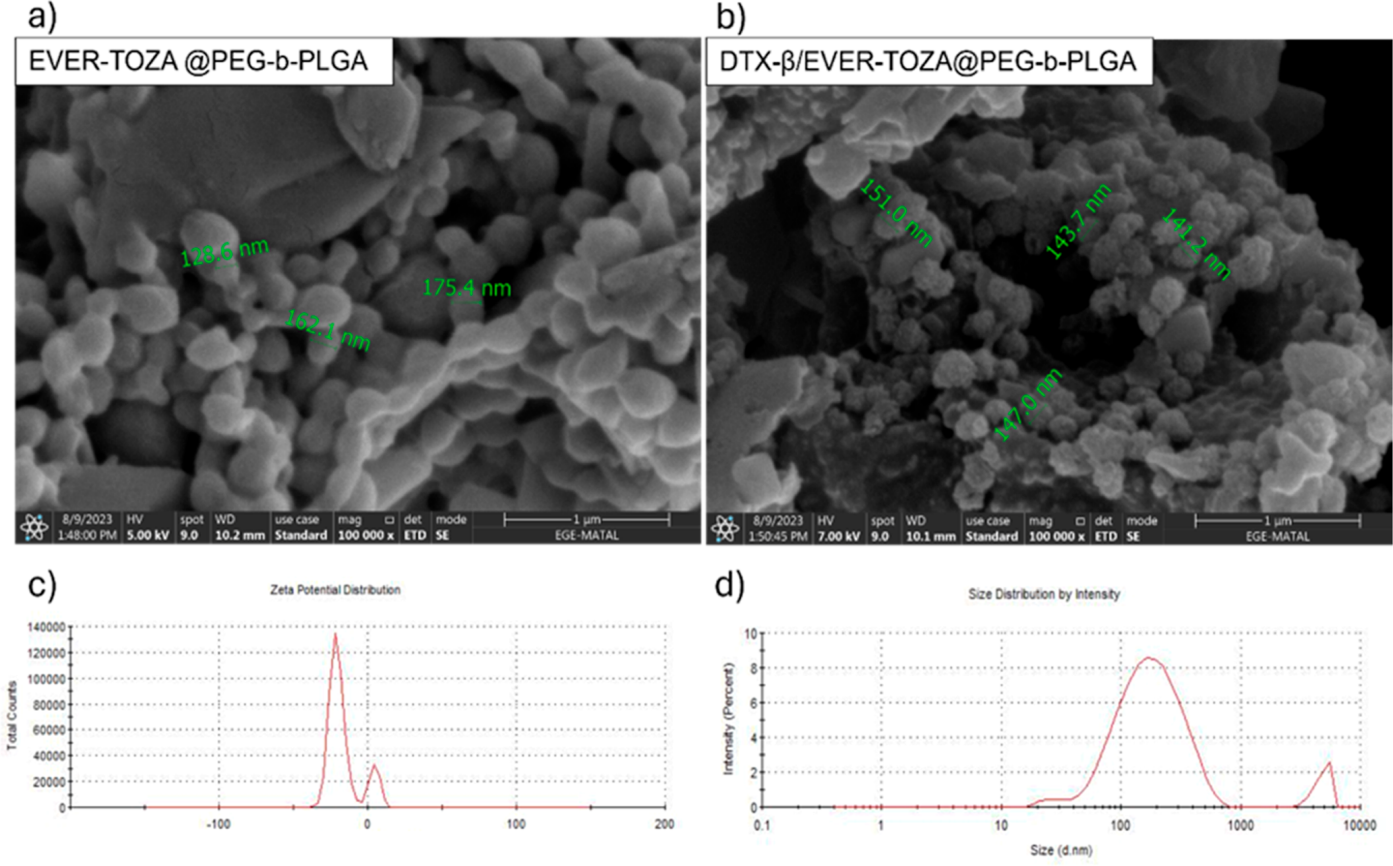
Structural Imaging of the NPs: SEM images (a,b) and DLS results (c,d) of EVER + TOZA@PEG-*b*-PLGA, and DTX-β/EVER + TOZA@PEG-*b*-PLGA NP.

SEM analysis showed spherical nanoparticles with sizes ranging approximately from 130 to 175 nm for EVER + TOZA@PEG-*b*-PLGA and 140 to 150 nm for DTX-β/EVER + TOZA@PEG-*b*-PLGA (Figure 1a,b). A detailed evaluation of the SEM micrographs showed that the DTX-β/EVER + TOZA@PEG-*b*-PLGA nanoparticles retained a smooth and spherical morphology. The slight increase in particle–particle proximity observed after DTX-β conjugation does not represent true aggregation but is a common appearance in PEG–PLGA systems with increased surface ligand density.^19^ Importantly, individual nanoparticle boundaries remained clearly distinguishable, and no fused clusters or continuous aggregates were detected. Similar observations have been reported for PEG–PLGA formulations containing high drug payloads, where mild surface interactions can occur without compromising colloidal stability.^20^ This interpretation is further supported by DLS measurements, which showed narrow size distributions and consistently negative zeta potentials, indicating stable and well-dispersed nanoparticles.

The sizes for this sample range from 140 to 155 nm, indicating a highly homogeneous size distribution.

For the DLS measurements, zeta potential values were obtained for both samples. The zeta potential for EVER + TOZA@PEG-*b*-PLGA was measured to be approximately −27 ± 5 mV (Figure 1c), indicating good stability. For DTX-β/EVER + TOZA@PEG-*b*-PLGA, the zeta potential was approximately −41 ± 1 mV, indicating even better stability. The size distribution graphs from the DLS measurements show that both samples have a small size distribution. The average size values, consistent with those obtained from the SEM images, were approximately 148 ± 2 nm for EVER + TOZA@PEG-*b*-PLGA and approximately 151 ± 7 nm for DTX-β/EVER + TOZA@PEG-*b*-PLGA (Figure 1c,d). As a result, SEM and DLS analyses indicate that both EVER + TOZA@PEG-*b*-PLGA and DTX-β/EVER + TOZA@PEG-*b*-PLGA NPs were successfully synthesized and characterized.

#### 3.1.3 FTIR Spectrum Results

The FTIR spectra presented in Figure 2 were recorded for the individual components (EVER, TOZA, DTX-β) as well as the formulated structures (EVER + TOZA@PEG-*b*-PLGA and DTX-β/EVER + TOZA@PEG-*b*-PLGA). The characteristic vibrational bands of PEG and PLGA were clearly observed in the nanoparticle spectra, including the aliphatic C–H stretching vibrations (2950–2850 cm^–1^), the ester carbonyl (C=O) stretching band of PLGA (1750–1700 cm^–1^), and the C–O–C and C–O stretching vibrations of PEG (1400–1000 cm^–1^).^30,31^ These signals confirm the integrity and incorporation of the PEG-*b*-PLGA copolymer matrix.

**Figure 2:**
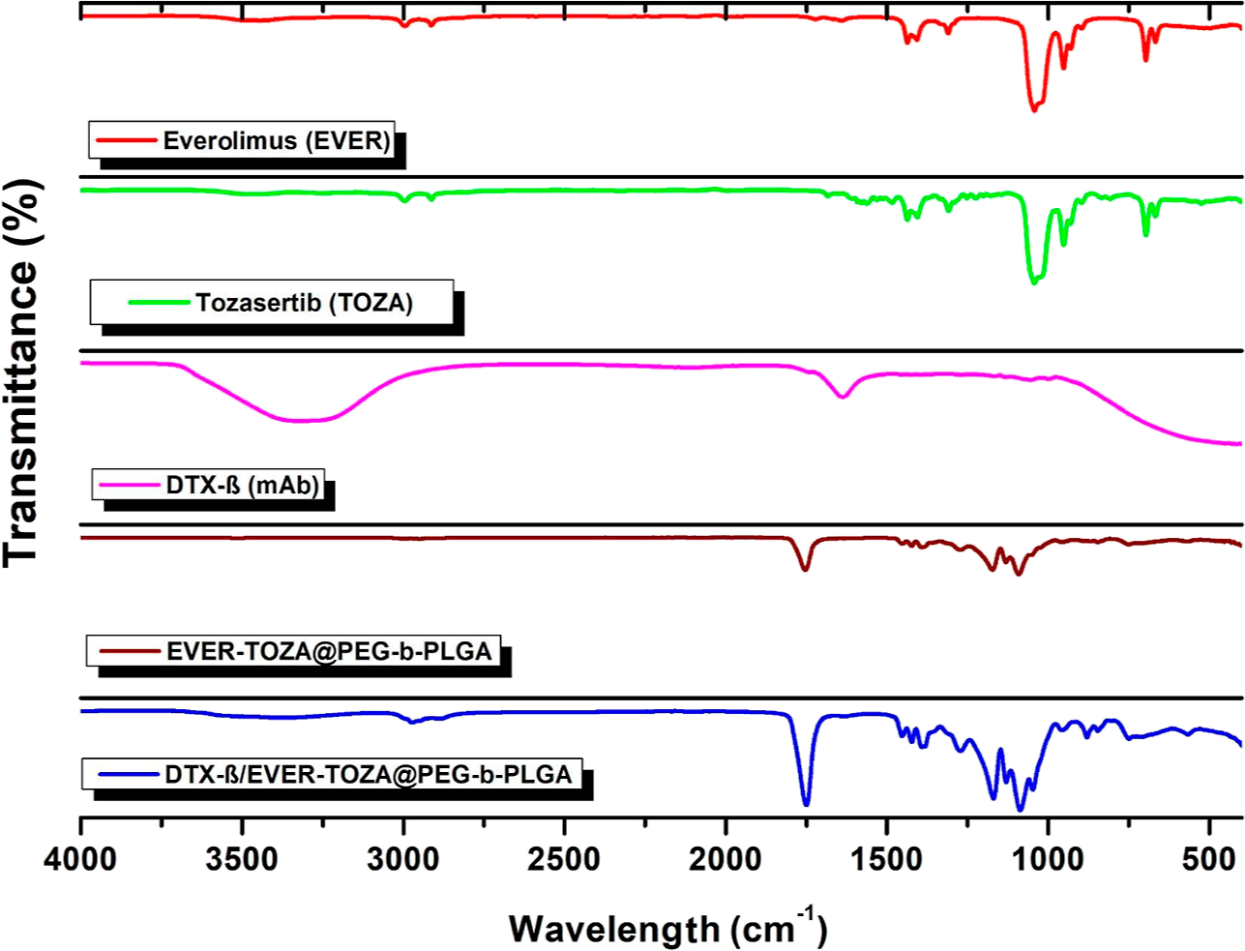
Structural detection of NPs: the ATR-FTIR spectra of EVER, TOZA, DTX-β, EVER-TOZA@PEG-*b*-PLGA, and DTX-β/EVER-TOZA@PEG-*b*-PLGA.

Drug-specific vibrational features were also evident. EVER exhibited aliphatic C–H stretching (2950–2850 cm^–1^), a carbonyl band near 1700 cm^–1^, and C–O/C–N stretching bands within 1400–1000 cm^–1^, while TOZA displayed aliphatic C–H stretching, aromatic C=C vibrations (1600–1500 cm^–1^), and characteristic C–N/C–O signals in the same region. DTX-β showed protein-related bands, including N–H stretching (3300–3000 cm^–1^), amide I (C=O stretching near 1650 cm^–1^), and amide II (N–H bending + C–N stretching around 1550 cm^–1^).^32,33^ These observations confirm the successful incorporation of the drugs into the polymeric matrix.

Importantly, comparison of the FTIR spectra before and after DTX-β conjugation reveals clear spectral modifications indicative of successful surface functionalization. In the EVER + TOZA@PEG-*b*-PLGA spectrum, the PLGA ester carbonyl peak (1750–1700 cm^–1^) and PEG-derived C–O–C vibrations dominate the polymer region. Following conjugation (DTX-β/EVER + TOZA@PEG-*b*-PLGA), the amide I band (∼1650 cm^–1^) becomes more pronounced and partially overlaps with the ester carbonyl region, while the amide II band (∼1540 cm^–1^) emerges distinctly. Additionally, the N–H stretching region (3300–3200 cm^–1^) shows a noticeable increase in intensity and broadening. These spectral changes—specifically, the enhancement of amide I/II bands and the modified carbonyl region—provide strong evidence for the successful covalent attachment of DTX-β to the nanoparticle surface. Although the spectral modifications observed by FTIR strongly support DTX-β attachment, FTIR cannot independently confirm covalent conjugation. Therefore, these findings are interpreted alongside BCA quantification, hydrodynamic size increase, and the more negative zeta potential values, which collectively confirm successful antibody functionalization.

### 3.2 Drug Loading

To ensure accurate quantification, calibration curves for EVER and TOZA were constructed and are now presented in Figure 3a. Both drugs displayed excellent linearity within the tested concentration range, with *R* ^2^ values of 0.9959 for EVER and 0.9773 for TOZA. Drug loading results of the PEG-*b*-PLGA nanoparticles are shown in Figure 3b. In the literature, EVER to the NPs loading rates are generally reported to range between 8% and 10%.^30^ These rates have been optimized to ensure controlled and sustained drug release from the NPs. For instance, NPs loaded with 8.8 ± 1.3% EVER have been effectively utilized in cancer therapy. In this study, EVER loading rate obtained was 30%, which is significantly higher than the rates reported in the literature. The term “loading rate” refers to the percentage of drug incorporated into the nanoparticles relative to the initial amount of drug used in the formulation process. A higher loading rate indicates that a greater proportion of the drug has been successfully loaded into the nanoparticle, which can improve therapeutic efficacy and extend the duration of drug release. This substantial increase in loading rate could enhance therapeutic efficacy and reduce dosing frequency. These findings demonstrate that PLGA–PEG NPs can efficiently load EVER and similar hydrophobic drugs, providing controlled release. Similarly, the loading rates of TOZA in NPs have been examined in the literature, with various formulations employed. Notably, in a study by Le et al., the loading efficiency of TOZA into PLA–TPGS (poly(lactic acid)–tocopheryl polyethylene glycol succinate) NPs was reported to be 76%.^34^ In this study, TOZA loaded NPs had an average size of 63 nm and a zeta potential of −30 mV. In our study, the loading rate of TOZA into PEG–PLGA NPs was found to be 50.2%. This rate is comparable to the TOZA loading rates reported in the literature. It should be noted that these drugs were loaded together into the nanomaterial. When expressing the loading rate, it can be specified as a 3:5 ratio (EVER/TOZA). These findings indicate that PEG–PLGA NPs can effectively load hydrophobic drugs such as EVER and TOZA, providing controlled release. This suggests that these NPs have significant potential for use in various therapeutic applications, particularly in cancer treatment.

**Figure 3:**
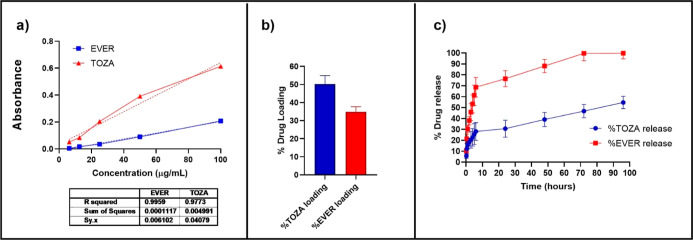
Calibration curve of EVER and TOZA (a) drug loading (b) and release (c) at pH 7.4 of EVER and TOZA from DTX-β/EVER-TOZA@PEG-*b*-PLGA NP.

### 3.3 In Vitro Drug Release

The amount of EVER and TOZA released from the DTX-β/EVER + TOZA@PEG-*b*-PLGA structure was determined separately. The release assay was performed in PBS pH 7.4 release buffers. Graphs plotted with data obtained from pH 7.4 release tests of DTX-β/EVER + TOZA@PEG-*b*-PLGA NPs are shown in Figure 3c. As a result, the release of drugs from the structure increased over time. In the release test conducted at pH 7.4, from the DTX-β/EVER + TOZA@PEG-*b*-PLGA structure, TOZA released approximately 30% at 24 h and about 55% of the drug at 96 h. EVER released approximately 76% of the drug at 24 h and 99% of the drug at 96 h from the structure. Release kinetics were fitted to zero-order, first-order, Higuchi, and Korsmeyer–Peppas models. The best correlation was obtained with the Korsmeyer–Peppas equation for both drugs (*R* ^2^ = 0.9606 for TOZA; *R* ^2^ = 0.9295 for EVER). The diffusion exponents (*n* < 0.45) confirm a Fickian diffusion mechanism. EVER exhibited a higher diffusion constant (*k* = 0.3482) compared to TOZA (*k* = 0.2725), consistent with its faster release from the PEG-*b*-PLGA matrix. In this release behavior, EVER was released faster from the structure compared to TOZA. The differing release rates of the two drugs are thought to be due to their molecular structures and the strength of their interactions with the PEG-*b*-PLGA nanoparticle matrix. EVER’s weaker interaction with the PEG-*b*-PLGA matrix may facilitate its faster diffusion into the aqueous environment, leading to a more rapid release. The hydrophobic nature of EVER is also considered to contribute to its easier diffusion into the looser regions of the matrix, resulting in faster release. In contrast, TOZA is assumed to have stronger interactions with the PEG-*b*-PLGA matrix, leading to its prolonged retention within the structure. The molecular size and solubility of TOZA are also significant factors affecting the release rate. These differences have led to distinct release profiles for the two drugs from the same matrix, with EVER exhibiting a faster release and TOZA showing a slower release profile. Similar findings in the literature support this hypothesis.^34,35^

### 3.4 In Vitro Cytotoxicity Assay

On 96-well plates, MTT measurements were performed at 24, 48, and 72 h. Different doses of DTX-β/EVER + TOZA@PEG-*b*-PLGA and EVER + TOZA@PEG-*b*-PLGA were administered to KELLY cells. The doses administered were 7.8 mg/mL, 3.9 mg/mL, 1.95 mg/L, 0.975 mg/L and 0.487 mg/mL. The combination of EVER and TOZA was administered at doses of 250 μM, 125 μM, 62.5 μM and 31.25 μM. EVER and TOZA were dosed at 2 mg/kg and 1.5 mg/kg, respectively. The 250 μM EVER + TOZA combination reported in the text corresponds to the in vitro equivalent concentration derived from these mg/kg doses.

The IC_50_ values at different concentrations of the drugs applied are shown in Figure 4. The IC_50_ value was recorded at 7.8 mg/mL in 24 h for DTX-β/EVER + TOZA@PEG-*b*-PLGA and EVER + TOZA@PEG-*b*-PLGA. The combination dose of the free-EVER + TOZA did not reach IC_50_ in 24 h. The lowest vitality rate in 48 h was 62 ± 2.3%. DTX-β/EVER + TOZA@PEG-*b*-PLGA and EVER + TOZA@PEG-*b*-PLGA NPs administered at a dose of 7.8 mg/mL significantly reduced cell viability compared to the control group (*p* < 0.05).

**Figure 4:**
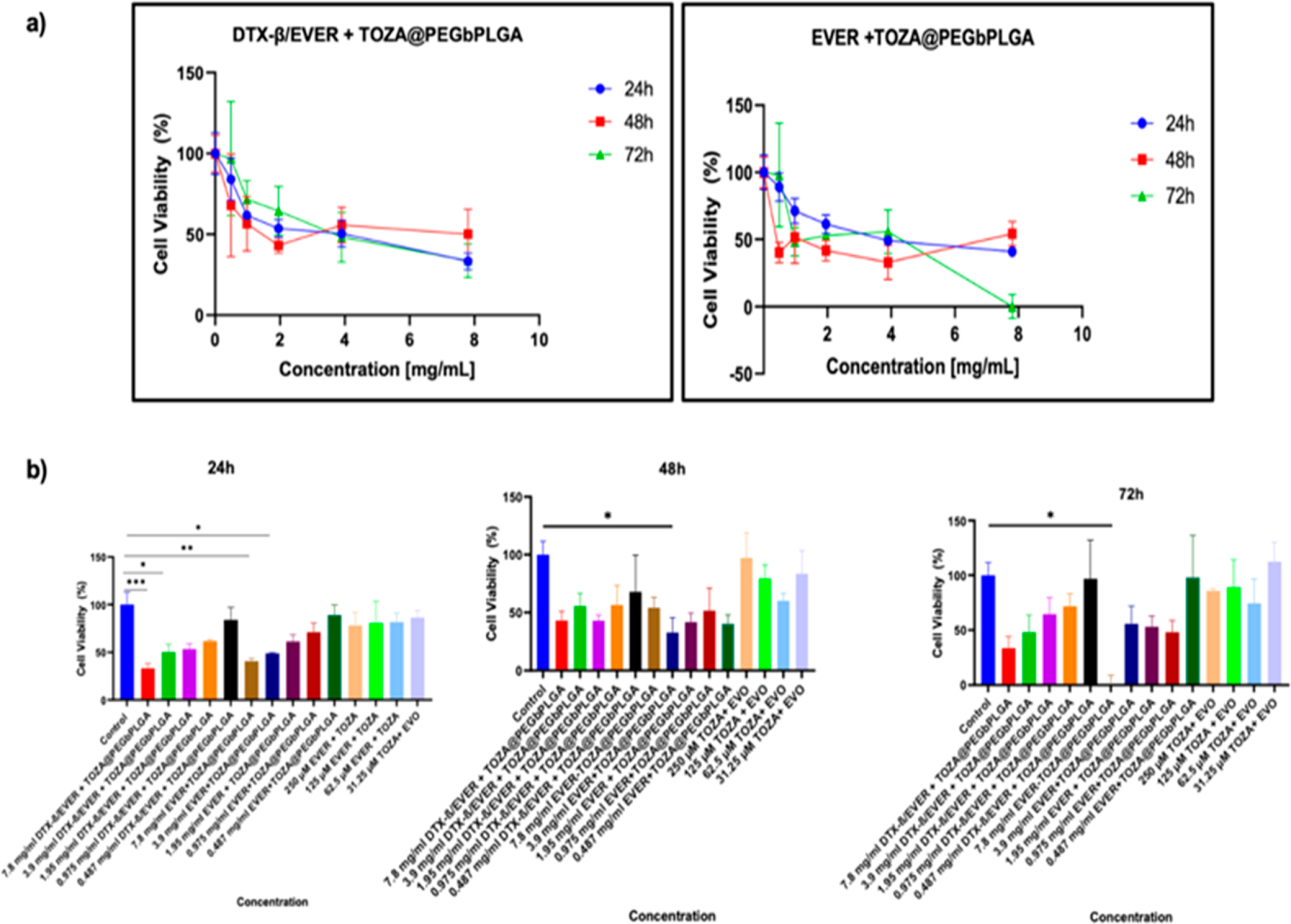
(a) IC_50_ values of the agents: Result of MTT analyses for KELLY cell line at different concentrations of DTX-β/EVER + TOZA@PEG-*b*-PLGA and EVER + TOZA@PEG-*b*-PLGA (*n* = 6). (b) 7.8 mg of DTX-β/EVER + TOZA@PEG-*b*-PLGA 7.8 mg of DTX-β/EVER + TOZA@PEG-*b*-PLGA showed a considerable reduction effect on cell viability within a 24 h period.

### 3.5 Flow Cytometry

Based on the results of the MTT analysis at KELLY, the cells were treated for 24 h with the IC_50_ concentration of EVER + TOZA@PEG-*b*-PLGA and DTX-β/EVER + TOZA@PEG-*b*-PLGA NP and free agents. Subsequently, cells were stained with annexin V and PI to assess the percentages of apoptotic and necrotic cells. The BD Accuri C6 software was used to calculate and analyze all flow cytometry data. According to our findings, the rates of early apoptosis had similar rates among the groups treated with DTX-β/EVER + TOZA@PEG-*b*-PLGA and EVER + TOZA@PEG-*b*-PLGA. Compared to control, there was an increase in apoptotic cells apoptotic cells were observed in 20.1% of the EVER + TOZA@PEG-*b*-PLGA group and 36.1% of the DTX-β/EVER + TOZA@PEG-*b*-PLGA group (Figure 5).

**Figure 5:**
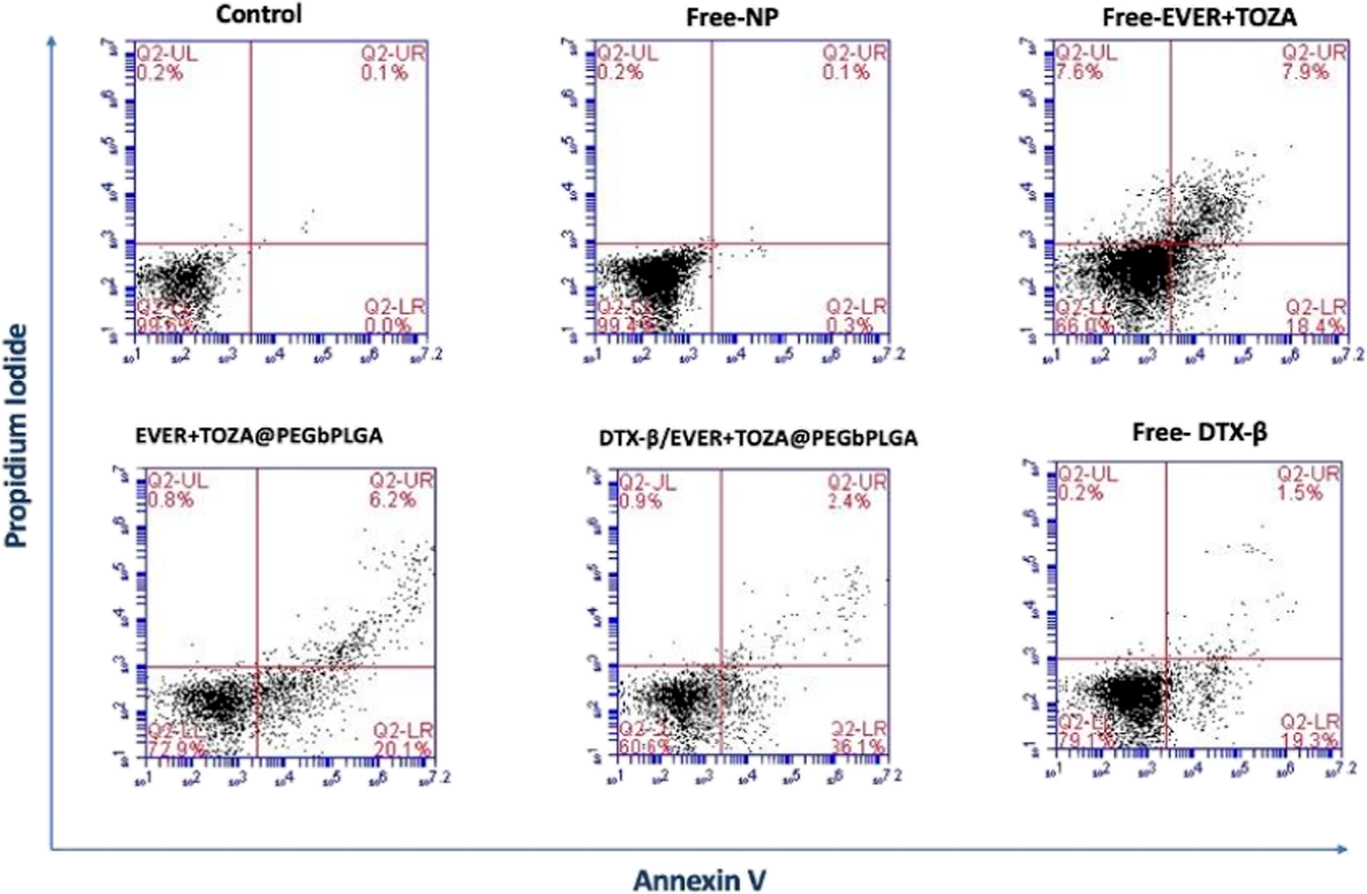
Evaluation of cellular death mechanisms: flow cytometry results of EVER + TOZA@PEG-*b*-PLGA and DTX-β/EVER + TOZA@PEG-*b*-PLGA NPs, free-EVER + TOZA combination, free NP and free-DTX-β. Q2-LL = live cells, Q2-LR = early apoptosis, Q2-UR = late apoptosis, O2-UL = necrosis.

In terms of vitality rate, the control and free-NP groups showed a similar pattern. About 99% of the cells were almost alive. We found that EVER + TOZA@PEG-*b*-PLGA showed lower necrosis rate in comparison to free-EVER + TOZA (Table 2). The encapsulation of EVER + TOZA in PEG-*b*-PLGA NPs likely due to better cellular uptake and sustained drug release.^36^ We observed an increased early apoptosis rates in the DTX-β/EVER + TOZA@PEG-*b*-PLGA by comparing with the EVER + TOZA@PEG-*b*-PLGA. Targeting of EVER + TOZA with an antibody leads to improved cell uptake, enhanced apoptosis, and reduced off-target effects.^37^

**Table 2:** Apoptosis/Necrosis Rates for the KELLY Cell Line

KELLY	% cell viability	% early apoptosis	% late apoptosis	% necrosis
control	99.6	0.0	0.1	0.2
free-NP	99.4	0.3	0.1	0.2
free-EVER + TOZA	66.0	18.4	7.9	7.6
EVER + TOZA@PEG-*b*-PLGA	72.9	20.1	6.2	0.8
DTX-β/EVER + TOZA@PEG-*b*-PLGA	60.6	36.1	2.4	0.9
free-DTX-β	79.1	19.3	1.5	0.2

### 3.6 In Vivo Tumor Growth Inhibition

KELLY cells with a MYCN amplification were studied in nude mice. DTX β/EVER + TOZA@PEG-*b*-PLGA and other agents were compared as treatment agents. 10^6^ KELLY cells were injected subcutaneously. Once the tumor size was 8 mm, the animals were divided into 5 groups: control, free-NP, free EVER + TOZA, EVER + TOZA@PEG-*b*-PLGA, and DTX-β/EVER + TOZA@PEG-*b*-PLGA. During the treatment, each agent was administered to a tail vein every day for 5 days. Then, animals were sacrificed. As shown in Figure 6, in the control group, the tumor size was about 18 mm. In the group treated with DTX-β/EVER-TOZA@PEG-*b*-PLGA, tumor sizes were recorded at an average of 5 mm. Statistical analysis revealed that tumor sizes were significantly reduced in all treatment groups (free-NP, free EVER + TOZA, EVER + TOZA@PEG-*b*-PLGA, and DTX-β/EVER + TOZA@PEG-*b*-PLGA) compared with the control group (Mann–Whitney *U*, *p* < 0.05 for all comparisons).

**Figure 6:**
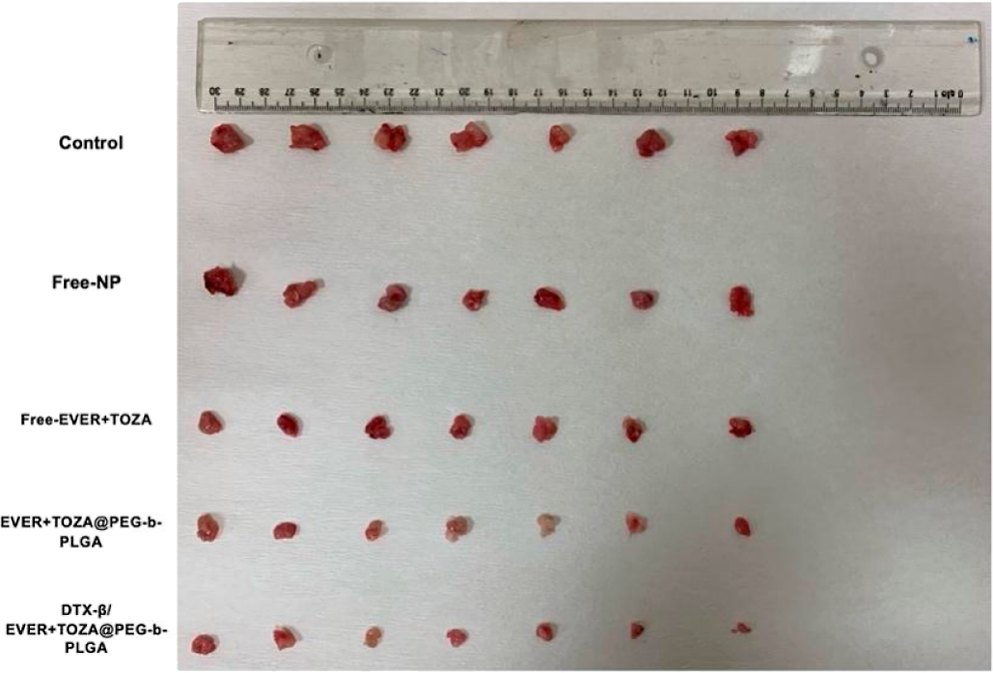
In vivo tumor size comparison: in the control group, the tumor size is over 10 mm. In the DTX-β/EVER-TOZA@PEG-*b*-PLGA-treated group, tumor sizes averaged 5 mm. A decrease in tumor size was observed in the groups treated with DTX-β/EVER-TOZA@PEG-*b*-PLGA. A reduction in tumor size was also observed in the EVER + TOZA@PEG-*b*-PLGA treatment group compared to the control. Representative tumors in each group are demonstrated (*n* = 7).

### 3.7 Effect of DTX-β/EVER + TOZA@PEG-*b*-PLGA Treatment on AURORA and MYCN Gene Expressions

RNA was obtained from mouse tumor tissues. After cDNA synthesis, qPCR was performed. Our findings showed an increase in MYCN and AURORA genes compared to the control group. MYCN mRNA levels in KELLY cells did not differ between free-EVER + TOZA and EVER + TOZA@PEG-*b*-PLGA treatment groups and the control. However, DTX-β/EVER-TOZA@PEG-*b*-PLGA increased mRNA expression.

Since the post-transcriptional processes are key to the final synthesis of the native protein, it cannot be assumed that the mRNA level it is directly correlated with its protein expression levels.^38^ As known in Figure 7, it is possible for there to be an over-abundance of mRNA relative to its corresponding protein in a cell. This can occur due to several mechanisms, including; translational regulation, mRNA stability, protein degradation, translational repression by miRNAs, post-transcriptional mechanisms, cellular stress or environmental conditions. EVER and TOZA may lead to increased transcription of certain genes as part of a feedback mechanism.^39^

**Figure 7:**
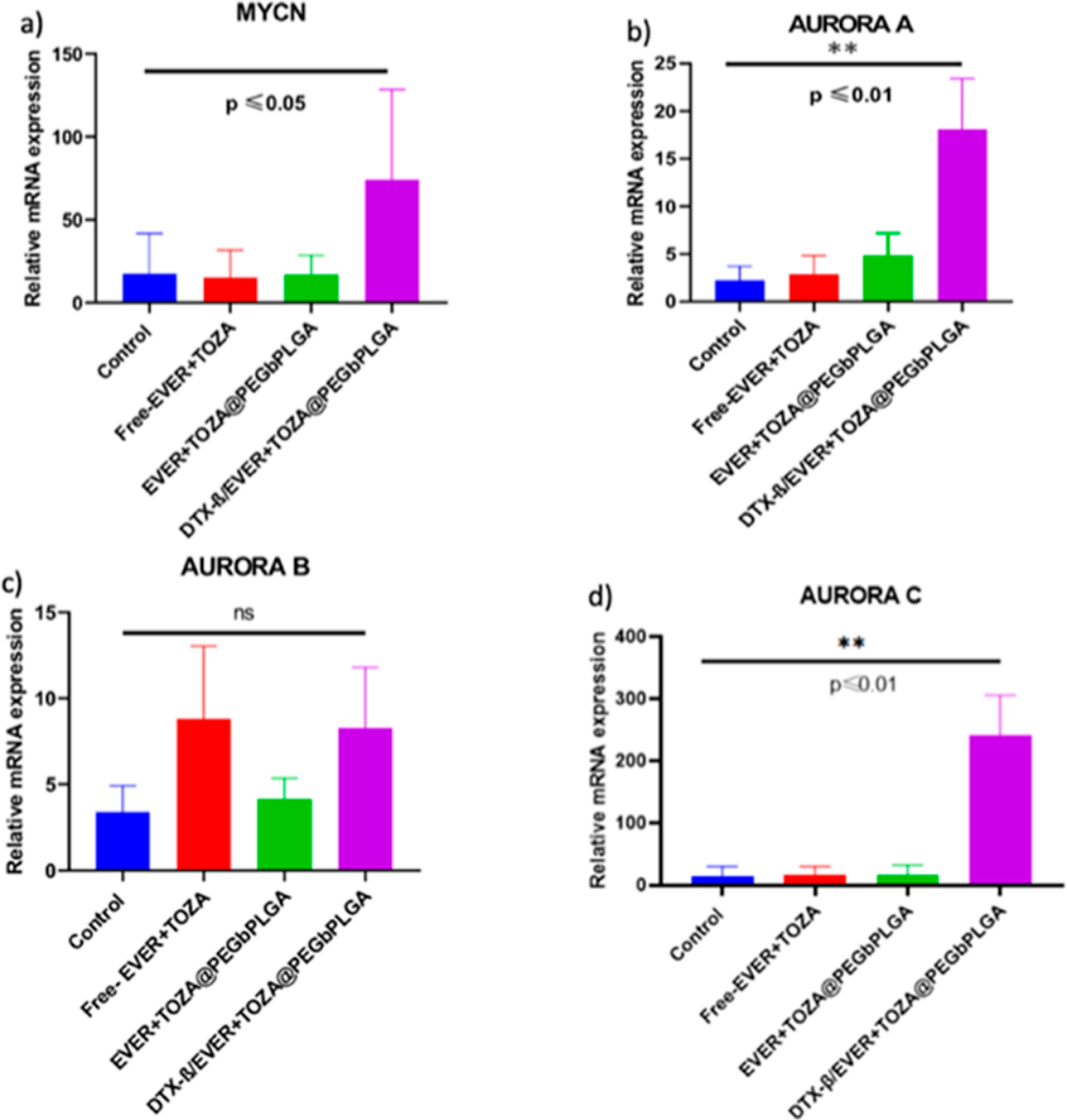
Relative mRNA expressions: MYCN mRNA levels in tumor tissues from xenograft mice (*n* = 7) generated with MYCN amplified KELLY cells were not different between the free-EVER + TOZA and EVER + TOZA@PEG-*b*-PLGA treatment groups and the control. However, DTX-β/EVER-TOZA@PEG-*b*-PLGA increased mRNA expression. (*p ≤ 0.05, **p ≤ 0.01). (a) MYCN mRNA expression. (b) AURKA mRNA expression. (c) AURKB mRNA expression. (d) AURKC mRNA expression.

### 3.8 Immunohistochemistry Based Protein Expression Determination

The expression of related proteins detected by IHC. After the treatment, mice were sacrificed. Tumor tissue was collected and prepared for histological analyses. As shown in Figure 8, protein levels for the control group demonstrated a high degree of positive MYCN and AURORA expression. AURORA and MYCN expression positivities were found to be low in the free-EVER + TOZA. DTX-β/EVER + TOZA@PEG-*b*-PLGA suppressed protein levels more than the EVER-TOZA@PEG-*b*-PLGA group.

**Figure 8:**
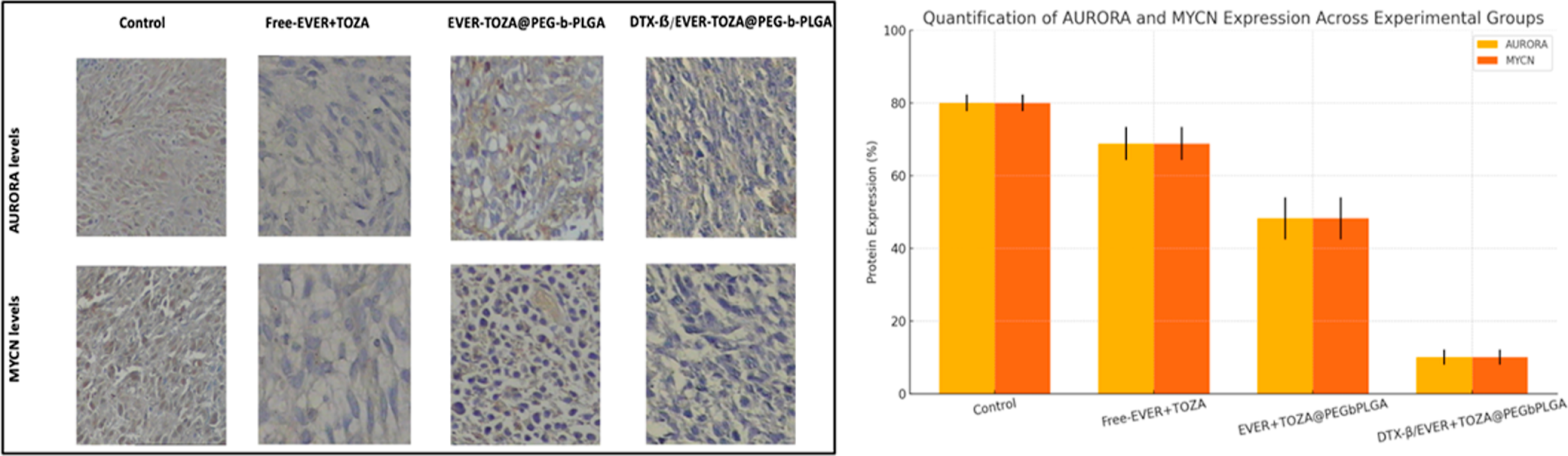
Protein expressions by IHC: MYCN and AURORA expressions in tumor tissues, magnification 20×. Tumor sections analyzed by immunohistochemistry. Control tissues with higher protein levels are used for comparison. In DTX-β/EVER + TOZA@PEG-*b*-PLGA group, the expression levels are suppressed more than the control, free-EVER + TOZA and EVER-TOZA@PEG-*b*-PLGA groups.

To determine the levels of apoptotic biomarkers, caspase 3, 8, and 9 antibodies treated with tumor tissues. As shown in Figure 9, caspase 3, 8, and 9 expression levels were significantly higher compared to the control group. The expression levels of caspase 8 and 9 were higher in the EVER + TOZA@PEG-*b*-PLGA and DTX-β/EVER + TOZA@PEG-*b*-PLGA groups, compared to the free-EVER + TOZA group.

**Figure 9:**
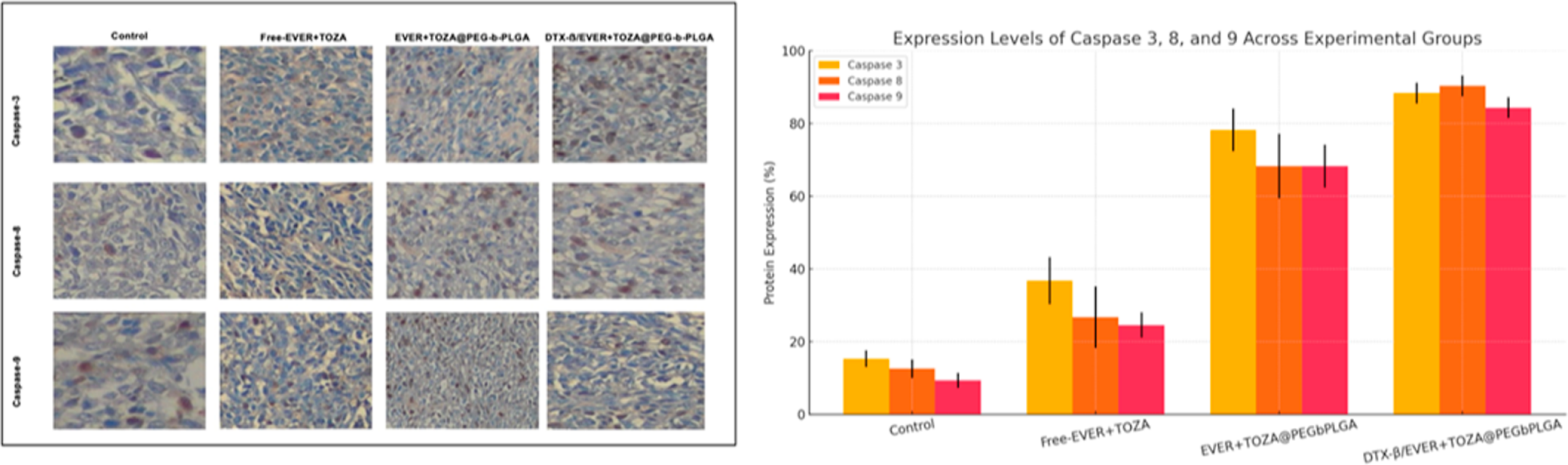
Apoptosis detection by IHC: caspase 3,8 and 9 expressions in tumor tissues, magnification 20×. Tumor sections analyzed by immunohistochemistry. The apoptotic biomarkers exhibited limited expression levels in the control group. The expression levels of caspase-3, caspase-8, and caspase-9 were found to be higher in the DTX-B/EVER + TOZA@PEG-*b*-PLGA and EVER + TOZA@PEG-*b*-PLGA groups.

Figure 10 shows immunohistochemical staining of GD_2_ in tumor sections derived from the Kelly neuroblastoma xenograft model following treatment with two distinct formulations. In the group treated with EVER + TOZA@PEG-*b*-PLGA (left panel), only limited GD_2_ staining was detected, suggesting modest intratumoral accumulation of the nontargeted nanoplatform. By contrast, the DTX-B/EVER + TOZA@PEG-*b*-PLGA group (right panel), in which the nanoplatform was conjugated with the anti-GD_2_ monoclonal antibody dinutuximab β, displayed markedly stronger DAB staining.

**Figure 10:**
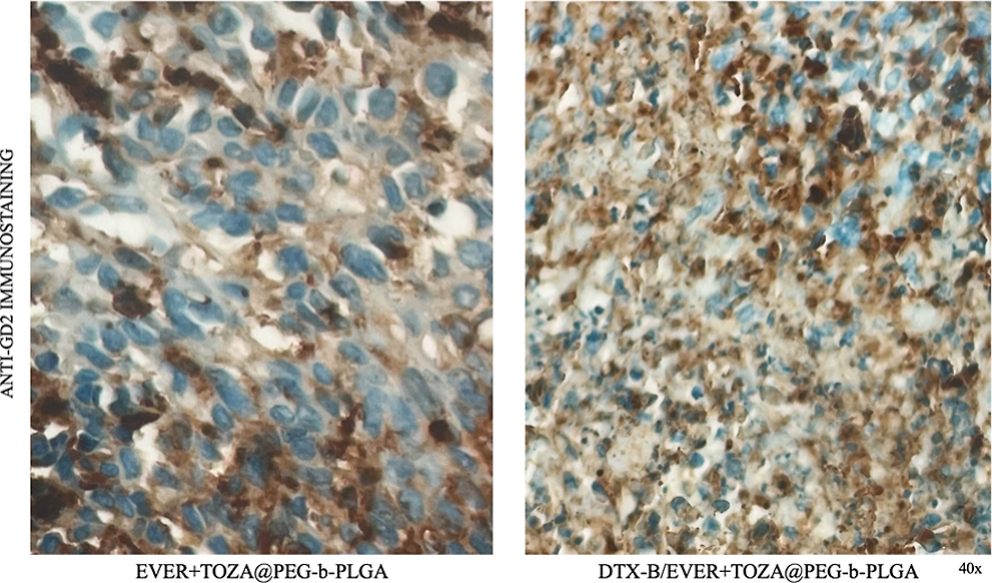
These histological findings are fully consistent with the in vivo therapeutic outcomes, where the DTX-B-functionalized formulation achieved the most pronounced tumor volume reduction. Collectively, these data demonstrate that GD_2_-directed functionalization enhances the tumor-targeting efficiency and intratumoral accumulation of the nanoplatform, thereby providing a mechanistic explanation for the superior antitumor efficacy observed in the xenograft model.

To objectively validate these observations, quantitative image analysis of multiple regions of interest (ROIs) was performed using QuPath software (QuPath 0.6.0-x64). The percentage of GD_2_-positive tumor cells was significantly higher in the DTX-B/EVER + TOZA@PEG-*b*-PLGA group (70.37 ± 14.62%) compared to the EVER + TOZA@PEG-*b*-PLGA group (53.28 ± 12.31%). Independent-samples *t*-test showed a trend toward increased positivity in the antibody-functionalized group (*t*(4) = −1.548, *p* = 0.197), and the calculated effect size was large (Cohen’s *d* = 1.26), supporting the biological relevance of this difference despite the limited sample size.

### 3.9 Histopathological Findings

Microscopic examinations of the kidneys from the treatment groups demonstrated normal function. All the groups showed typical hepatic configurations, including the shape of lobules, components of the portal hepatic space, and hepatocytes. When all tissues were examined, a slight infiltration of tumor cells in the kidney was observed in the control and free-NP group (Figure 11). However, the kidneys in the treatment groups had no abnormality. The free-NP, EVER + TOZA@PEG-*b*-PLGA, and DTX-β/EVER + TOZA@PEG-*b*-PLGA groups did not show any indications of liver toxicity. Spleen tissues were normal in the NP-treated groups. There were no histological differences determined in terms of cardiotoxicity between the groups.

**Figure 11:**
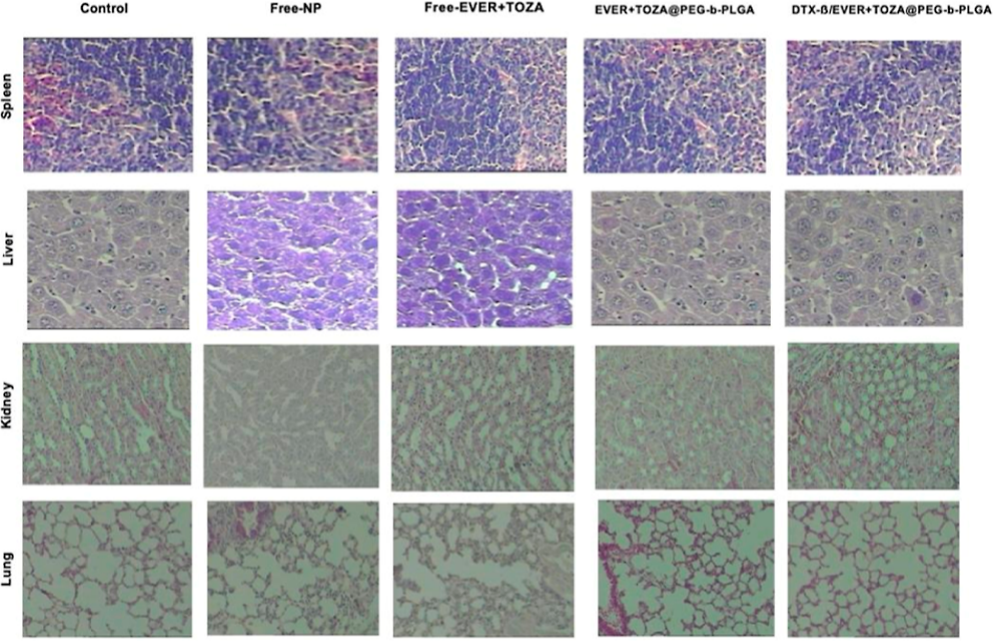
Detection of toxicity at the tissue level: histopathological view of the spleen, liver, kidney, lung and cardiac tissues belonging to both control and treatment groups. H&E staining, 20×.

## 4 Discussion

NPs exhibit a smooth and spherical morphology, a small size distribution, and high stability as indicated by their zeta potentials.^40,41^ These data confirm that the NPs are suitable for effective drug delivery and controlled release. Studies have shown that PEG–PLGA NPs generally exhibit spherical morphology and smooth surfaces, similar to the findings in our SEM images.^32,33^ The small size distribution observed in our study is consistent with previous research, where PEG–PLGA NPs typically show a narrow size range, ensuring efficient cellular uptake and enhanced biodistribution.^43^ The high zeta potential values observed in our study indicate good colloidal stability, which is essential for prolonged circulation time and reduced aggregation in biological systems. Similar zeta potential values have been reported for PEG–PLGA NPs in the literature, supporting their stability and suitability for drug delivery applications.^31,32^ Additional literature further corroborate these findings. For instance, studies by Marinelli et al. have reported similar size and zeta potential values for PEG–PLGA NPs, highlighting their stability and efficiency in drug delivery systems.^42^ Similarly, Mares et al. have emphasized the tunability of PLGA–PEG NPs, noting their excellent biocompatibility and controlled release properties, which align with our findings.^18^

The initial burst release is a common feature in NP drug delivery systems and has been observed in multiple studies.^44,45^ This behavior is advantageous for achieving immediate therapeutic effects followed by prolonged drug action. The burst release can be attributed to the rapid release of drug molecules located near the NP surface, while the sustained release is due to the gradual degradation of the PLGA matrix and diffusion of the drug.^20^ For instance, a study by Danhier et al. reported a similar initial burst release followed by a controlled release for drugs encapsulated in PLGA NPs.^31^ This can be advantageous for achieving a high initial therapeutic concentration of the drug. The sustained release observed in the current study is also supported by previous findings. For example, Zhang et al. demonstrated that PLGA-based NPs provide a sustained release of encapsulated drugs over several days to weeks, depending on the polymer composition and drug properties.^20^ The controlled release helps in maintaining therapeutic drug levels over an extended period, reducing the frequency of administration. The release profiles of hydrophobic drugs like EVER and TOZA encapsulated in PEG–PLGA NPs are consistent with the release behavior observed for other hydrophobic drugs. Studies have shown that the encapsulation efficiency and release kinetics are highly dependent on the hydrophobicity of the drug and the polymer–drug interactions.^33^

The impact of PEGylated NPs on organ toxicity is a subject of challenging investigation. This is attributed to their ability to reduce immune recognition and prolong circulation time.^46^ Indeed, many research has demonstrated that PEGylated NPs are well-tolerated, with minimal adverse effects on these organs. However, the safety profile can depend on factors like the size, surface charge, and dosage of the nanoparticles.^47–49^ Our study shown that the PEG-*b*-PLGA based NPs have no adverse effects on organ function when evaluated in vivo. The free-NP group did not exhibit any signs of organ toxicity. Only the infiltration of tumor cells through the kidney was observed. Since the tumor cells were injected subcutaneously when the in vivo model was generated, in the nontreated groups (control and free-NP) tumor growth had progressed and adhesion to part of the kidney. The lack of tumor infiltration into the kidney in the treatment groups may be considered as an evidence of the drugs’ effectiveness in reducing tumor growth.

EVER is an mTOR inhibitor. It is well-known that mTOR signals actively control transcription factors in cells necessary for proliferation.^50,51^ TOZA is a pan-AURORA kinase inhibitor. AURORA and mTOR inhibition can lead to increased transcription of certain genes as part of a feedback mechanism.^52^ We found that there was an increase in MYCN and AURORA at the mRNA level in our investigation. However, there was a decrease in protein level. This apparent discrepancy between increased mRNA and decreased protein expression has been widely described in contexts where mTOR or AURORA pathways are pharmacologically inhibited. mTOR blockade suppresses cap-dependent translation, reduces ribosomal biogenesis, and limits global protein synthesis, preventing increases in transcription from being translated into higher protein levels.^54^ Moreover, AURORA kinase inhibition destabilizes MYCN by enhancing proteasomal degradation, thereby shortening MYCN protein half-life.^11^ Thus, although feedback-driven transcriptional upregulation occurs, the combined EVER + TOZA treatment simultaneously diminishes translational capacity and accelerates protein turnover.^55^ This dual mechanism provides a biologically coherent explanation for why MYCN and AURORA mRNA levels increase while their protein abundance declines in our study.

Vaughan et al. similarly demonstrated destabilization of MYCN by inhibiting protein expression through mTOR inhibition.^52^ In our investigation, we also verified that EVER reduces MYCN at the protein level. Michaelis et al. demonstrated that drug-resistant NB cells treated with TOZA had an increase in apoptotic cell death.^53^ Our flow cytometry findings support these results. Le et al. showed the apoptosis/necrosis rates of NP-encapsulated and free forms of TOZA.^33^ Similar to our findings, necrosis was observed in the free-EVER + TOZA treated group, but in encapsulated group, there was an increase in the rate of apoptosis and a decrease in the rate of necrosis.

## 5 Conclusion

In conclusion, DTX-β/EVER-TOZA@PEG-*b*-PLGA has decreased MYCN protein levels. In NB treatment, targeted nanocarriers may provide a more potent cytotoxic and apoptotic effect directly to the tumor site. mTOR and AURORA inhibitors can be encapsulated within the PEG-*b*-PLGA NP structure specifically targeted to the region expressing the tumor-specific antigen Anti-GD_2_ monoclonal antibody. DTX-β/EVER-TOZA@PEG-*b*-PLGA shows antitumoral properties against NB, achieving it through mechanisms of apoptosis and necrosis. Antibody targeting of EVER + TOZA increases cellular uptake, promotes apoptosis, and diminishes off-target effects.

In our study, we intend to evaluate the NP encapsulating mTOR and AURORA inhibitors, directed toward the tumor region using anti-GD_2_, in NB cell lines exhibiting diverse clinical features. Simultaneously, it is essential to do toxicity assessments on the synthesized NP. It is essential to perform escalating dose toxicological investigations on experimental animals, including BALB/C mice, Wistar albino rats, and rabbits. Research on NP targeting utilizing various chemotherapeutic drugs, such as cisplatin, may be conducted. In this context, further studies and clinical trials are needed.

## Supplementary Material



## Notes

BSA standard calibration curve used for DTX-β quantification by BCA assay (Figure S1) and UV–vis standard calibration curves of EVER and TOZA (Figure S2) (PDF)
