# Supplementary material for: Role of Anti-GD2 Targeted PEG‑b‑PLGA Nanoparticles in the Treatment of MYCN Driven Neuroblastoma
Source: ACS Appl Bio Mater. 2026 Apr 2;9(8):3753–67. doi: 10.1021/acsabm.5c01709 (PMC13102200; doi:10.1021/acsabm.5c01709)
Supplement: Supplementary file 1 [file mt5c01709_si_001.pdf]

Supporting Information

The Role of Anti-GD2 Targeted PEG-b-PLGA Nanoparticles in the Treatment of MYCN Driven Neuroblastoma

Ozde Gokbayrak [1], Derya Ozel [2], Ayca Tuncel [2], Fatma Yurt [2], Hatice Efsun Kolatan [3], Aylin Erol [1], Efe Ozgur Serinan [1], Tekincan Aktas [1], Osman Yilmaz [3], Safiye Aktas [1*]

[1] Department of Basic Oncology, Institute of Oncology, Dokuz Eylul University, Izmir 35340, Turkey

[2] Department of Nuclear Applications, Institute of Nuclear Sciences, Ege University, Izmir 35100, Turkey

[3] Department of Laboratory Animal Science, Institute of Health Sciences, Dokuz Eylul University 35340, İzmir, Turkey

* Correspondence: safiye.aktas@deu.edu.tr

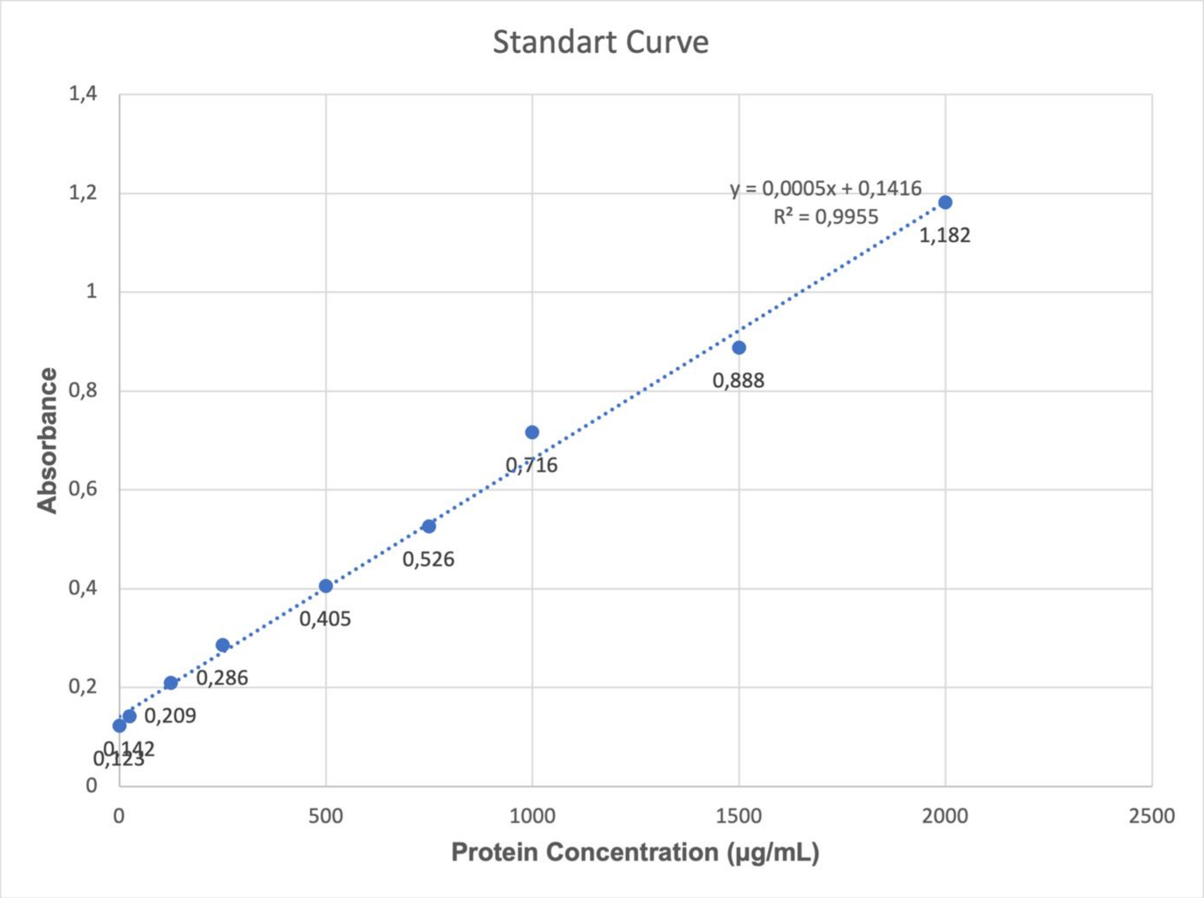

Figure S1. BSA standard calibration curve used in the BCA assay.

Absorbance at 562 nm plotted against BSA concentration. Linear regression equation and R^2 are indicated.

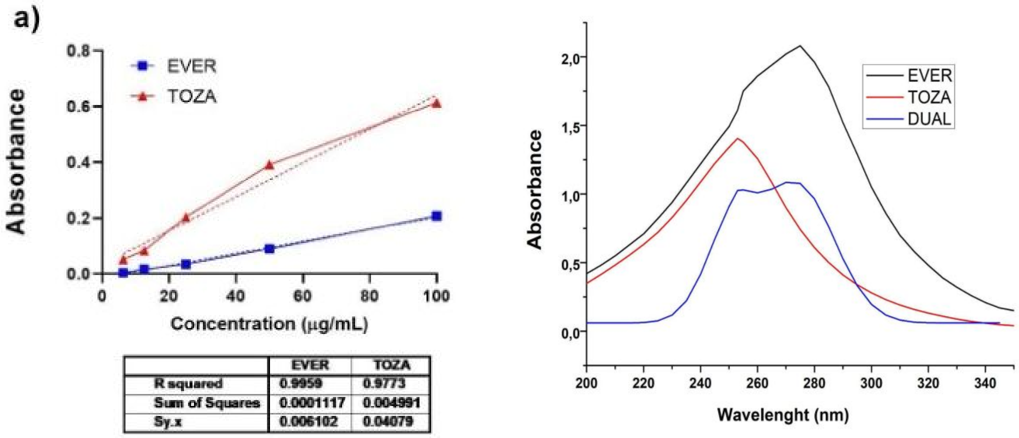

	EVER	TOZA
R squared	0.9959	0.9773
Sum of Squares	0.0001117	0.004991
Sy.x	0.006102	0.04079

Figure S2. UV/Vis standard calibration curves of EVER and TOZA.

UV/Vis absorbance spectra of EVER (blue) and TOZA (red) measured at their respective detection wavelengths across increasing concentrations (0–100 µg/mL). Linear regression curves, R^2 values, sum of squares, and Sy.x values are provided in the table below.
